# Machine learning-based lifetime breast cancer risk reclassification compared with the BOADICEA model: impact on screening recommendations

**DOI:** 10.1038/s41416-020-0937-0

**Published:** 2020-06-22

**Authors:** Chang Ming, Valeria Viassolo, Nicole Probst-Hensch, Ivo D. Dinov, Pierre O. Chappuis, Maria C. Katapodi

**Affiliations:** 1 Department of Clinical Research, Faculty of Medicine, University of Basel, Basel, Switzerland; 2 Oncogenetics and Cancer Prevention, Geneva University Hospitals, Geneva, Switzerland; 3 Swiss Tropical and Public Health Institute, University of Basel, Basel, Switzerland; 4 Department of Computational Medicine and Bioinformatics, University of Michigan, Ann Arbor, MI USA; 5 Michigan Institute for Data Science, University of Michigan, Ann Arbor, MI USA; 6 Statistics Online Computational resource, University of Michigan, Ann Arbor, MI USA; 7 University of Michigan School of Nursing, Ann Arbor, MI USA; 8 Genetic Medicine, Geneva University Hospitals, Geneva, Switzerland

**Keywords:** Cancer screening, Population screening

## Abstract

**Background:**

The clinical utility of machine-learning (ML) algorithms for breast cancer risk prediction and screening practices is unknown. We compared classification of lifetime breast cancer risk based on ML and the BOADICEA model. We explored the differences in risk classification and their clinical impact on screening practices.

**Methods:**

We used three different ML algorithms and the BOADICEA model to estimate lifetime breast cancer risk in a sample of 112,587 individuals from 2481 families from the Oncogenetic Unit, Geneva University Hospitals. Performance of algorithms was evaluated using the area under the receiver operating characteristic (AU-ROC) curve. Risk reclassification was compared for 36,146 breast cancer-free women of ages 20–80. The impact on recommendations for mammography surveillance was based on the Swiss Surveillance Protocol.

**Results:**

The predictive accuracy of ML-based algorithms (0.843 ≤ AU-ROC ≤ 0.889) was superior to BOADICEA (AU-ROC = 0.639) and reclassified 35.3% of women in different risk categories. The largest reclassification (20.8%) was observed in women characterised as ‘near population’ risk by BOADICEA. Reclassification had the largest impact on screening practices of women younger than 50.

**Conclusion:**

ML-based reclassification of lifetime breast cancer risk occurred in approximately one in three women. Reclassification is important for younger women because it impacts clinical decision- making for the initiation of screening.

## Background

Breast cancer is the most common malignancy affecting women worldwide and the fifth leading cause of cancer death.^1^ In Switzerland, about 6000 women are diagnosed with breast cancer each year, and more than 1350 die from the disease.^2^ Most established risk factors, i.e., age, family history, genetic predisposition, hormone and reproductive factors and history of benign breast disease, are not applicable for primary prevention to reduce breast cancer incidence and mortality.^3^ Survival of breast cancer patients in the past few decades has mostly improved through screening, especially if tumours are diagnosed at early stages, and through advances in therapeutic approaches.^3–5^ Breast cancer remains a public health problem, and early detection is currently the best option to reduce its impact.

Breast cancer screening with biennial mammograms for women 50–74-years old has been recommended by the U.S. Preventive Services Task Force since 2009.^6,7^ In Europe, nationally organised screening programmes began around 1985 in the Nordic countries and the United Kingdom, followed by other European countries.^8,9^ Most of these programmes target women from 50 to 69 years old for screening.^10^ In 1995, the Swiss Federal Office of Public Health and the Swiss Cancer League adopted a national programme recommending biennial mammography screening for women over 50 years old.^2,11^ In 2013, the Swiss Cancer League adopted the UK NICE Clinical Guidelines, which recommend screening with mammography and MRI based on women’s risk classification. The guidelines classify women into moderate (17% ≤ lifetime risk <30%) or high (lifetime risk ≥30%) breast cancer risk calculated with the BOADICEA model based on different scenarios of family history.^12,13^

Age over 50 years is the sole risk factor considered for entering a population-based screening programme.^14^ However, about 25% of all breast cancers are diagnosed in younger women.^15,16^ Moreover, mammography is less effective as a screening tool for younger women, who are more likely to have dense breast tissue, compromising the efficiency of routine mammograms in this age group. This contributes to diagnostic delays and increased morbidity and mortality.^16,17^ In the era of personalised medicine, a screening strategy based on individual breast cancer risk may improve the benefit–harm ratio of mammography, and increase the efficiency of screening programmes.^18,19^ Many medical societies and professional groups proposed that risk-based screening would be more effective, less morbid and more cost-effective.^3,19–24^

Although many models are used to predict breast cancer risk, such as the Breast Cancer Risk Assessment Tool (BCRAT, also referred as the Gail model), the International Breast Intervention Study (IBIS) model, the Breast and Ovarian Analysis of Disease Incidence and Carrier Estimation Algorithm (BOADICEA model),^25–27^ no consistent model has been incorporated into routine clinical practice and/or screening programmes due to limited discriminatory accuracy and applicability. The discriminatory ability, area under the receiver operating characteristic (AU-ROC) curve, of these models is between 0.53 and 0.64.^26,28–31^ A comprehensive risk prediction model with an improved discriminatory power to classify women into clinically meaningful risk groups will enable targeting high-risk women, while reducing interventions in those at low risk.

Machine-learning (ML) algorithms offer an alternative approach to standard prediction modelling that may address current limitations and improve the accuracy of breast cancer prediction models.^32,33^ A series of ML techniques, including our own work, have been developed and used in breast cancer prediction and prognosis, demonstrating that the application of ML methods could improve the prediction accuracy of cancer susceptibility, recurrence and survival models.^34–39^ Previous studies presented the discriminatory accuracy, sensitivity, specificity and calibration performance of different ML algorithms. However, clinical utility, in terms of potential clinical consequences of using new ML prediction models, is rarely examined. The objective of the current study is to assess the impact of using ML-based breast cancer risk prediction on screening practices. Using data from a large clinical population, we quantified performance measure and reclassification of lifetime breast cancer risk generated from ML algorithms and from the BOADICEA model. We also examined the clinical impact of reclassification of breast cancer risk on screening practices based on the Swiss Surveillance Protocol.^13^

## Methods

### Swiss clinic-based retrospective data

The Oncogenetic Unit at the Geneva University Hospitals has been offering genetic counselling and testing for hereditary cancer syndromes since 1994 to patients and asymptomatic individuals concerned with their family history. The common reasons for genetic consultation are familial aggregation of breast or colorectal cancer or suspicion of hereditary cancer syndromes, mainly due to breast, ovarian or colon cancer. For each individual seen in consultation, demographic, personal and family history, previous genetic test results and a detailed family pedigree are collected and recorded with the ‘Progeny' software.^40^ Data used in this study were collected as part of routine medical records. The Regional Research Ethics Committee at the University Hospitals of Geneva has approved the data collection and management processes. Informed consent was obtained from all participants included in the study before genetic testing.

For the purposes of this study, information regarding pathology reports, archived tumour tissue and cancer treatment, was extracted from medical records for cancer patients and affected relatives, whenever possible. Data from genetic consultation records and Progeny files were extracted with R packages ‘tm’ and ‘gdata’.^41^ Extracted data were suitable for risk calculations with the BOADICEA model for multiple female members from each family. There were about 13% missing values. *BRCA1/BRCA2* status, oestrogen receptor and progesterone receptor status contributed 11%. In addition to ‘positive' and ‘negative', missing values for *BRCA* pathogenic variants and hormone receptor testing were characterised as ‘unknown' in subsequent analyses. This approach is also consistent with the flexibility of the BOADICEA model in handling missing information.

### BOADICEA model classification

Lifetime risk predictions were generated with the web-based batch processing from the BOADICEA web application (version 3.0) using data from 2481 families with 112,587 family members. The lifetime breast cancer risk for each woman in every family was calculated using data from all other family members, and by assigning every female family member once as the targeted woman for risk calculation.

### ML risk classifications

We generated breast cancer lifetime risk predictions for all female members within each family.

Based on previous reports of method reliability, effectiveness and performance in identifying, tracking and exploiting salient features in similar samples with the same data structures, we selected three ML algorithms, i.e., Markov Chain Monte Carlo generalised linear mixed model (MCMC GLMM),^42^ adaptive boosting (ADA) and random forest (RF).^32,34,42–45^ The input for the ML algorithms used identical risk factors as the ones included in the BOADICEA model in order to have fair comparisons among the different risk prediction models. The variables included in each comparison are presented in Supplementary Table 1.

In our supervised classification, we rebalanced the breast cancer patients and cancer-free controls to reduce potential bias with the R packages ‘unbalanced' (Racing for Unbalanced Methods Selection) and ‘SMOTE' (Synthetic Minority Over-sampling TEchnique).^46,47^ SMOTE implements known ML techniques to adaptively select the most appropriate strategy for a given unbalanced task. To ensure the reliability of ML predictions and the consistency of the forecasts, we used 10-fold cross-validation with 20 repetitions. This strategy provides a powerful preventive measure against model overfitting.^48–50^

### Comparisons of performance measure and classification

BOADICEA cannot be applied for females older than 80 years, for males and for deceased individuals. Thus, we excluded all predictions generated for those individuals when we compared the performance of ML algorithms with the BOADICEA model. The performance of BOADICEA was evaluated from *n* = 45,110 women using the AU-ROC, while the performance of ML techniques is presented with the mean AU-ROC from 10-fold cross-validations.

According to the Swiss Surveillance Protocol, we applied the following cut-offs for lifetime breast cancer risk: <17% as near-population risk, ≥17% and < 30% as moderate risk and ≥30% as high-risk group. We excluded women who were under 20-years old or had been diagnosed with breast cancer to be consistent with the clinical utility of the protocol. We estimated differences in breast cancer risk classification using the BOADICEA model and the best-performing ML algorithm, based on the data from *n* = 36,146 breast cancer-free women.

### Statistical analyses

Frequencies, percentages, means and standard deviations were used to describe the demographics and clinical characteristics of 36,146 breast cancer-free women. We present classifications by age and risk categories using the BOADICEA model as the reference standard. Differences in classification for mammography surveillance according to the Swiss Surveillance Protocol were calculated for the moderate- and high-risk groups.

## Results

**Fig. 1:**
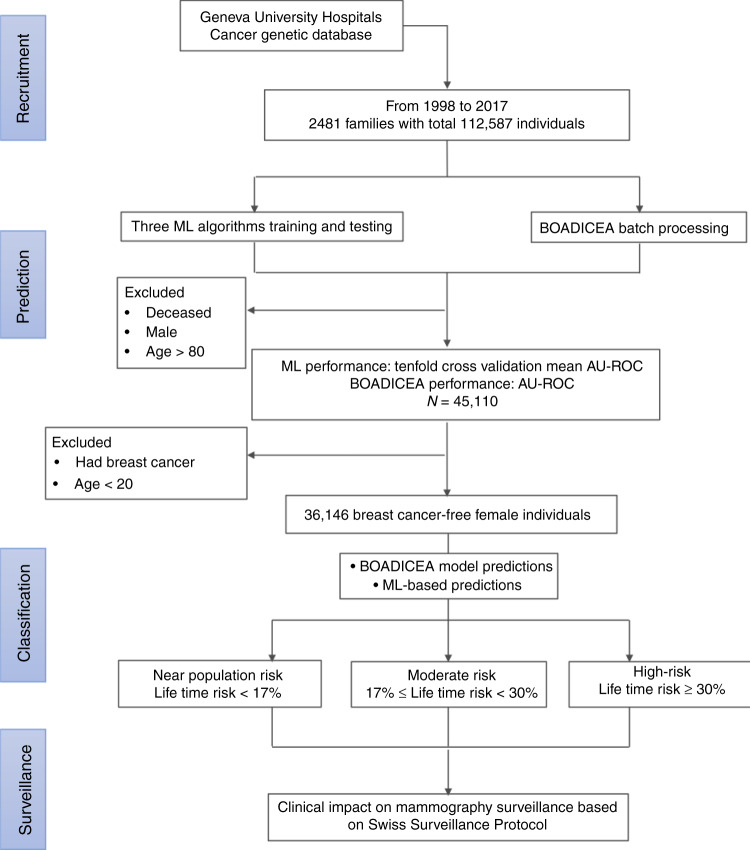
Consort flow diagram of the whole cohort with breast cancer risk-based classification. ML machine learning, BOADICEA Breast and Ovarian Analysis of Disease Incidence and Carrier Estimation Algorithm, AU-ROC Area Under the Receiver Operating Characteristic curve.

A consort flow diagram (Fig. 1) presents sample acquisition, prediction, classification and surveillance status, and the overall process of methodology and materials.

### Model performance

**Fig. 2:**
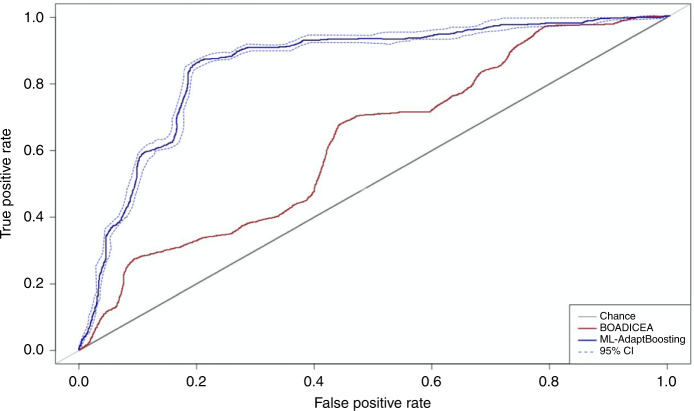
Receiver operating characteristic (ROC) curves of the ML-adapt boosting and BOADICEA model predicting breast cancer lifetime risk, *N* = 45,110 female individuals. ML machine learning, BOADICEA Breast and Ovarian Analysis of Disease Incidence and Carrier Estimation Algorithm, CI confidence interval.

The mean age of the 45,110 women was 49.82 (±11.02) years old. There were 4911 breast cancer patients with average age onset at 51.76 (±9.79) years old. Among them, 554 had a second breast cancer diagnosis. There were 119 cases with first-ductal carcinoma in situ (DCIS). Table 1 presents the performance comparison of the three ML algorithms compared with the BOADICEA model. Using the same risk factors, the accuracy of ML techniques was superior to the BOADICEA model for the Swiss clinic-based samples. Predictive accuracy reached 88.9% using ADA, 85.1% using MCMC GLMM and 84.3% using RF versus 63.9% using the BOADICEA model, showing an approximately 20–25% increase in accuracy. Figure 2 presents the ROC curves that visualise the accuracy improvement between the BOADICEA model and ADA, which was the best-performing ML approach.

**Table 1:** Performance by area under the receiver operating characteristic curve (AU-ROC) of the machine-learning (ML) algorithms predicting breast cancer lifetime risk derived from 10-fold cross-validations compared with the BOADICEA model.

Algorithms	AU-ROC	Standard deviation	95% Confidence interval		Absolute change from BOADICEA
			LCL	UCL	
BOADICEA	0.639	–	–	–	–
ML-ADA	0.889	0.005	0.885	0.903	+25.0%
ML-MCMC GLMM	0.851	0.006	0.847	0.856	+21.2%
ML-RF	0.843	0.008	0.838	0.849	+20.4%

*MCMC GLMM* Markov Chain Monte Carlo generalised linear mixed model, *ADA* adaptive boosting, *RF* random forest, *LCL* lower confidence limit, *UCL* upper confidence limit. *BOADICEA* Breast and Ovarian Analysis of Disease Incidence and Carrier Estimation Algorithm.

*N* = 45,110 female individuals.

### Breast cancer-free women

Table 2 presents demographic and clinical characteristics of the Swiss clinic-based sample. Among *n* = 36,146 breast cancer-free women, 2617 (7.24%) had a diagnosis of another type of cancer. In the total sample, only few breast cancer-free women (462; 1.3%) were tested for *BRCA1* and/or *BRCA2* germline pathogenic variants, including both complete and targeted testing. Most of these women had a targeted genetic testing, i.e., the search for a pathogenic variant previously identified in the family, since consultations were limited to situations that are highly suggestive for a hereditary syndrome and, whenever possible, genetic testing was offered first to breast cancer patients belonging to the family.

**Table 2:** Characteristics of the breast cancer-free female cohort.

Demographics and clinical characteristics	Breast cancer-free female cohort, *N* = 36,146 (% or ± SD)
Age (years)	51.09 ± 15.35
Age at menarche (years)	12.82 ± 1.51
Age at first live birth (nulliparous excluded, years)	24.10 ± 5.03
Parity (nulliparous excluded)	1.92 ± 1.32
Age at menopause (premenopausal women excluded, years)	47.94 ± 6.68
Ashkenazi Jewish ancestry	239 (0.66%)
Ethnicity (Black)	828 (2.29%)
BRCA1 or BRCA2 germline pathogenic variants	115 (462 tested)
Cancer diagnosis (all types)	2 617 (7.24%)
Age at cancer onset (years)	57.44 ± 15.96
Colorectal cancer	574 (1.59%)
Age at colorectal cancer onset (years)	61.63 ± 17.19
Lung/bronchus cancer	153 (0.42%)
Age at lung/bronchus cancer onset (years)	62.01 ± 26.18
Pancreatic cancer	136 (0.38%)
Age at pancreatic cancer onset (years)	66.85 ± 22.94
Ovarian cancer	508 (1.40%)
Age at ovarian cancer onset (years)	55.96 ± 22.84

*N* = 36,146 individuals.

### Classification comparison

When using the BOADICEA model as the reference standard, and based on the lifetime breast cancer risk cut-offs from the Swiss clinical guidelines, 58.8% of all samples were categorised as near-population risk, 32.3% as moderate risk and 8.8% as high risk (Table 3). Compared with the BOADICEA model, ML-ADA classified 7968 women into the high-risk group, which is an increase of 4790 samples. ML-ADA also classified 16,465 women into near-population risk group, which is a decrease of 4818 samples compared with the BOADICEA model. Concordance between the BOADICEA model and ML-ADA was ~60% in the near-population and the moderate-risk groups, while it was 87.95% in the high-risk group. ML-ADA classified 9595 women (26.55%) to a higher- risk group and 3174 (8.78%) women to a lower-risk group. When we combined Table 3 with the Swiss Surveillance Protocol, we identified an additional 2469 (14.83%) women younger than 50 who needed early-onset screening.

**Table 3:** Comparisons of lifetime risk classification between ML-Adapt Boosting (ML-ADA) algorithm and the BOADICEA model (reference standard) for the breast cancer-free cohort.

Risk age	Near-population risk BOADICEA risk < 17%, *N* = 21,283			Moderate risk 17% ≤ BOADICEA risk < 30%, *N* = 11,685			High-risk BOADICEA ≥ 30%, *N* = 3178		
	ML-ADA < 17%	17%≤ ML-ADA < 30%	ML-ADA ≥ 30%	ML-ADA < 17%	17%≤ ML-ADA < 30%	ML-ADA ≥ 30%	ML-ADA < 17%	17%≤ ML-ADA < 30%	ML-ADA ≥ 30%
20–29 (*n* = 4 959)	2181	430	215	372	1050	233	17	41	420
30–39 (*n* = 5 277)	2069	645	430	407	989	256	18	34	429
40–49 (*n* = 6 410)	2466	832	625	442	1191	326	20	44	464
50–59 (*n* = 7 025)	2681	899	751	535	1243	337	25	49	505
60–69 (*n* = 6 436)	2037	745	849	570	1326	349	23	43	494
70–80 (*n* = 6 039)	2116	871	441	465	1233	361	21	48	483
Total	13,550	4422	3311	2791	7032	1862	124	259	2795
Concordance	63.67%	–	–	–	60.18%	–	–	–	87.95%
Reclassification	**–**	20.78%	15.56%	23.89%	**–**	15.93%	3.90%	8.15%	**–**

– Does not apply.

*N* = 36,146. ML machine learning, BOADICEA Breast and Ovarian Analysis of Disease Incidence and Carrier Estimation Algorithm, ADA adaptive boosting.

### Clinical impact on mammography surveillance

Table 4 presents the overall number differences in mammography surveillance when applying the BOADICEA and ML-ADA models, and based on the Swiss Surveillance Protocol. For women 40–59 years old, ML-ADA grouped an additional 184 women in the moderate-risk group, suggesting annual mammography surveillance. ML-ADA grouped an additional 4,790 women in the high-risk group, among which 2535 women were between 30 and 59 years old, suggesting annual mammography, and 1865 women older than 60 years, suggesting biennial mammography.

**Table 4:** Clinical impact on mammography screening based on Swiss Surveillance Protocol.

*ML* machine learning, *BOADICEA* Breast and Ovarian Analysis of Disease Incidence and Carrier Estimation Algorithm, *ADA* adaptive boosting.

## Discussion

We used a novel approach to identify individuals at increased risk of breast cancer by using ML algorithms. We analysed family history, cancer pathology and clinic–demographic data from a large retrospective dataset of *n* = 112,587 individuals from 2481 families. We examined whether ML algorithms could improve predictive accuracy for breast cancer compared with the BOADICEA model. We also quantified the differences in risk classification and the impact on screening between these two techniques based on the Swiss Surveillance Protocol. Compared with the BOADICEA model, all three ML techniques were superior at distinguishing cancer cases from cancer-free women, and improved the predictive accuracy by 20–25% using exactly the same risk factors as the BOADICEA model. These findings clearly demonstrate the inherently better predictive ability of ML algorithms.

About one in four women were classified into a higher-risk group compared with the BOADICEA model. Given that ML approaches achieved much higher discriminatory accuracy, some women’s breast cancer risk would have been underestimated when using the BOADICEA model, while one in eleven women’s risk would have been overestimated. When taking into account the Swiss Surveillance Protocol, the major discordance for mammography surveillance was observed for the high-risk group. About 10–15% women 30–80 years old were underscreened when using the BOADICEA model compared with ML-ADA.

Consistent with other national screening programmes, the Swiss national breast cancer screening programme is based on age alone, starting at 50 years old. This approach will miss some breast cancers in moderate- and high-risk women 40–49 years old and in high-risk women 30–49 years old. The development and implementation of risk-based breast cancer control and prevention strategies have important public health implications. Common risk estimation models, like the BOADICEA model, are currently used in clinical practice to provide evidence for adjustment of screening, i.e., more frequent mammographic screening and initiation at a younger age. However, low discriminatory accuracy has greatly limited the clinical utility of these models. At the population level, ML algorithms have reached high sensitivity and can be implemented to identify high-risk women who should initiate earlier breast cancer screening. At the individual level, the decision for preventive interventions, such as prophylactic mastectomy or use of tamoxifen as a risk-reducing agent, is influenced by a woman’s individualised breast cancer risk estimate. When using ML, one in three women were classified into different risk categories compared with the BOADICEA model, which may lead to different preventive interventions.

Given that breast cancer screening guidelines were established after the release of several commonly used risk prediction models, including BOADICEA, the guideline cut-offs (risk categorisation) have been greatly influenced by these models. According to several validation studies of the BOADICEA model, about 80–90% of women were classified as having a lifetime breast cancer risk between 5 and 25% (near population or moderate risk).^25^ This risk distribution was also observed in our study. However, using a 17% cut-off within a non-disperse risk distribution may have resulted in low discriminatory accuracy for women around that cut-off (17% or ‘near population risk'). When we reclassified women with ML algorithms, applying cut-offs of 17% and 30% resulted in shifting relatively large proportions of women between different risk groups. This indicates that for ML algorithms, categorisation of different risk groups (i.e., near population, moderate or high risk) should be probably based on different cut-offs, based on a clinically meaningful decision of their sensitivity and specificity.^51^

There are several barriers for using risk prediction models in a wide variety of settings. First, each risk prediction model uses different risk factors. The panel of risk factors used in the development of each model limits its applicability and validity in broader segments of the population. ML models can be applied in medical consultation contexts where similar data inputs were collected. Currently, the most feasible way of following the Swiss Surveillance Protocol is through consultation with a medical specialist. In this context, clinical decisions about risk management options are likely influenced by risk calculations from such prediction models. Secondly, existing infrastructures for collection and assessment of clinical data limit the development of risk prediction models and their generalisability in broader segments of the population. ML approaches have the potential to achieve better accuracy, and can incorporate different types of information, including mammographic images, family history, germline genetic data and clinical factors. However, currently there are no comprehensive systems that incorporate data from such diverse sources, e.g., screening programmes, medical consultations and medical records. In order to develop a risk prediction model that can be used to enhance national screening programmes, the usefulness of accessible risk factors from screening practice, e.g., breast density and previous benign breast disease, should be assessed. Based on the predictive ability of each risk factor, and the feasibility of collecting relevant data in the screening setting, a parsimony panel of risk factors would be applied in ML modelling to develop a comprehensive model that supports effective clinical decision-making. However, limited resources have been invested into this promising new analytic approach.

### Strengths and limitations

Our results are reliable because we used a limited number of well-established breast cancer risk factors without feature selection and relatively non-complex ML models, which helps mitigate the ‘black-box' nature of ML algorithms. They are also reliable due to the large sample size, completeness and high accuracies of the data. Our models have been evaluated for internal validity, since we have reproduced similar accuracy performance in this study compared with our previous study.^34^ They have been partially evaluated for external validity using internal statistical cross-validation, a process where each fold iteration relies on separate and independent training and testing datasets. For fully assessing the external validity, we need to evaluate prospective samples from populations intrinsically different from the development sample, in respect to location, time or methods/criteria used for data collection, which is a gradual process commonly applied to prediction models.^26,30^ Current screening guidelines already incorporate risk estimates from existing prediction tools based on inputs from medical consultation contexts. Thus, it is important to study the potential clinical utility of ML as a promising alternative analytic approach, even with limited information from screening practice. Finally, breast cancer surveillance guidelines define ‘population level risk' as having a lifetime risk <17% calculated from the BOADICEA model. This risk estimate does not necessarily mean ‘low' risk in the general population due to potential misclassifications. In our reclassification results, the BOADICEA model classified 21,293 (58.9%) samples into the population-level risk. Thus, our sample is ‘suitable' for the comparison and covers sufficiently women with a wide range of risk estimates based on current recommendations.

One limitation of the study is that the performance of our approaches was evaluated with k-Fold cross-validation process in the same dataset, which could result in an optimistic model performance. However, the k-Fold cross-validation process generally results in a less biased or less optimistic estimate of the model skill compared with other commonly used methods, e.g., simple train/test split.^52^ Moreover, we used retrospective cross-sectional data, which limit the ability of ML algorithms to generate 5- or 10-year risk estimates. Analysing prospective longitudinal data with ML algorithms may reveal additional implications for clinical decision support.

In summary, we calculated lifetime breast cancer risk with ML algorithms and compared their discriminatory accuracy, classification and impact on mammography screening with the BOADICEA model according to the Swiss Surveillance Protocol. Differences in classification and impact on breast cancer surveillance were considerable. The ability of our model to detect individuals with high suspicion of breast cancer, should be further evaluated with other datasets and prospective samples. Future studies can enhance the performance of ML algorithms through incorporation of additional clinical data, such as lifestyle, medications, breast images, exact histology of benign breast diseases and co-morbidities.^36,37,53^ Future studies can also include resource rearrangement involving health policymakers and other stakeholders, in terms of cost-effectiveness and adaptability in different clinical settings. A prospective clinical trial would provide more functional and extended evaluation of the performance of ML algorithms, and findings can be compared with ongoing personalised breast cancer screening trials like ‘My PeBS' and ‘WISDOM'.^54,55^

## Supplementary information

Supplementary Table 1

## Data Availability

The datasets used and analysed during the current study are available from the corresponding author upon reasonable request and gaining signed clinical data transfer agreement from Geneva University Hospital. We also shared the computational protocol via GitHub (https://github.com/SOCR/ML_BCP/).
