# Supplementary material for: Machine learning-based lifetime breast cancer risk reclassification compared with the BOADICEA model: impact on screening recommendations
Source: Br J Cancer. 2020 Jun 22;123(5):860–7. doi: 10.1038/s41416-020-0937-0 (PMC7463251; doi:10.1038/s41416-020-0937-0)
Supplement: Supplementary file 1 — Supplementary Table 1 [file 41416_2020_937_MOESM1_ESM.docx]

**Supplementary Table 1.** Input variables in the AI/ML and BOADICEA models.

| Variables list | Variables used in BOADICEA model | Variables used in ML-based model |
| --- | --- | --- |
| Family pedigree (beyond 2^nd^ degree relatives contained affected and unaffected members from both maternal and paternal side) including: | ✓ | ✓ |
| Age (or age at death) | ✓ | ✓ |
| Gender | ✓ | ✓ |
| Vital status | ✓ | ✓ |
| Ashkenazi Jewish ancestry | ✓ | ✓ |
| Ovarian cancer diagnosis and age of onset | ✓ | ✓ |
| Prostate cancer diagnosis and age of onset | ✓ | ✓ |
| Pancreatic cancer diagnosis and age of onset | ✓ | ✓ |
| Breast cancer diagnosis and age of onset | ✓ | ✓ |
| Contralateral breast cancer diagnosis and age of onset | ✓ | ✓ |
| Estrogen receptor status (for breast cancer only) | ✓ | ✓ |
| Progesterone receptor status (for breast cancer only) | ✓ | ✓ |
| HER2 status (for breast cancer only) | ✓ | ✓ |
| BRCA/BRCA2 germline pathogenic variant | ✓ | ✓ |
